# Alcohol dependence: international policy implications for prison populations

**DOI:** 10.1186/1747-597X-1-33

**Published:** 2006-11-08

**Authors:** Gail Yvonne Jones, Norman G Hoffmann

**Affiliations:** 1 Rehabilitation for Addicted Prisoners Trust U.K., 39 Wellington Street, Thame, Oxfordshire, 0X9 3BW, UK; 2 Evince Clinical Assessments, 29 Peregrine Place, Waynesville, NC 28786, USA

## Abstract

**Background:**

In light of the emphasis on drug abuse, this study explored the relative prevalence of substance use disorders among United Kingdom (UK) prison inmates in the context of findings from a general inmate population in the United States (US). The lead author of the report conducted a structured diagnostic interview with 155 new admissions to one of two prisons in the UK using the CAAPE (Comprehensive Addiction And Psychological Evaluation), a structured diagnostic interview, to ensure consistent assessments. The US sample consisted of 6,881 male inmates in a state prison system evaluated with an automated version of the SUDDS-IV (Substance Use Disorder Diagnostic Schedule-IV) interview.

**Results:**

Alcohol dependence emerged as the most prevalent substance use disorder in both UK prisons and in the US sample. Relative frequencies of abuse and dependence for alcohol and other drugs revealed that dependence on a given substance was more prevalent than abuse ad defined by the current diagnostic criteria.

**Conclusion:**

Despite the emphasis on drugs in correctional populations, alcohol dependence appears to be the most prominent substance use disorder among the incarcerated in both the US and UK and must be considered in developing treatment programs and policy priorities.

## Background

While correctional systems have been conscious of the relationship between alcohol use disorders and crime [1] they have traditionally focused on providing treatment intervention for prisoners whose crimes are drug related. While both the United States (US) and the United Kingdom (UK) have developed National Strategies supported by significant levels of funding to address the problems of illicit drug use, there remains a conspicuous absence of priorities in addressing the social and economic consequences of alcohol related crime other than those involving driving.

Some drug treatment approaches have also underestimated the potential importance and implications of untreated alcohol dependence. While some studies such as those of Caputo and colleagues [2] indicated a reduction in alcohol use amongst individuals on a short-term methadone programme, studies have shown that individuals on a longer term methadone maintenance programme self-report a significant increase in alcohol consumption [3,4]. The historical emphasis on the treatment of illicit drug use and substitute prescribing may have failed to recognize that a significantly high percentage of individuals addicted to opiates and other drugs are also addicted to alcohol. In a study into the level of alcohol consumption among clients in methadone maintenance Hillebrand and colleagues reported that 51% of methadone patients met the DSM-1V criteria for alcohol dependence [5].

Previous findings on inmate populations in the United States have documented that alcohol dependence as defined by the DSM-IV and DSM-IV-TR [6,7] is the most prevalent substance use disorder [8]. These previous analyses found that not only is alcohol dependence more prevalent than dependence on other substances, it also emerges as more prevalent than alcohol abuse in inmate populations when detailed diagnostic interviews are employed. Interestingly, general indications of dependence appear to be more prevalent than abuse only among adjudicated adolescents in secure facilities [9].

While research has shown that it is difficult to demonstrate a clear causal relationship between alcohol and violent crime, the British Medical Association has estimated that either the offender or victim has consumed alcohol in 65% of homicides, 75% of stabbings, 70% of assaults and half of all domestic assaults. In the UK it has been estimated that 78% of assaults are committed under the influence of alcohol. The Prime Minister's Strategy Unit's Interim Analysis estimates that there are 1.2 million incidents of alcohol related violence, 360,000 alcohol related incidents of domestic violence and 85,000 cases of drink-driving per annum in the UK [10].

The primary purpose of the study is to determine whether findings are compatible across different assessment instruments and procedures. As mentioned previously, earlier reports have shown alcohol dependence to be more prevalent than abuse or other dependence diagnoses in correctional populations in the United States. The question addressed is whether similar patterns emerge using a different detailed diagnostic interview in prisons where the probability of behavioural health conditions is high.

## Methods

### Samples

The British sample consisted of 155 men representing consecutive admissions to correctional institutions at HMP Grendon (n = 118) and consecutive referrals to the RAPt drug treatment programme at HMP Aylesbury (n = 37) who were interviewed during the calendar year 2003. The interviews utilized the CAAPE (Comprehensive Addictions And Psychological Evaluation), a structured interview based on the DSM-IV/DSM-IV-TR criteria for substance use and mental health disorders. A structured interview was chosen to ensure consistent questioning.

Grendon, as with other UK prison estates, is run by a prison service governor; however, it operates a unique regime in its therapeutic care of offenders through six wings operating as autonomous therapeutic communities. The prison is designated a category B prison, many of whose inmates are serving life sentences. All prisoners transferred to the establishment must have a minimum of two years remaining on their sentence to be able to complete the therapeutic process offered.

Aylesbury houses young male offenders aged between 18 and 21. In 1989 Aylesbury was designated as a long-term young offender institution holding some of the longest sentenced young adult males in the English prison system.

Overall, the ages of the inmates ranged from 18 to 66 years on admission with an average of 30.7 (SD = 9.05) and a median of 31. Approximately 80% were white; 10% identified themselves as black either of African, Caribbean, or other decent; and 3% identified themselves as Asian. The remaining inmates were of other or mixed ethnic backgrounds. Sixty-eight percent had never married and only 5% were married at the time of incarceration. Forty-four percent had no educational qualifications and only 40% had been employed prior to incarceration. Of the sample, 69% reported that their offence was either alcohol or drug related.

Male Minnesota state prison inmates between the ages of 18 and 65 provided a comparison sample of 6,881 cases. Although the US sample differed in the ethnic mix, other demographic characteristics were similar to the UK sample. Only 51% of the US sample where white and African-Americans (31%), Native Americans (8%), and Hispanics/Latinos (7%) represented the largest ethnic groups. The average age was 30.8 years (SD = 9.28) and the median age is 29.0. As with the UK sample, approximately 68% had never married. Thirty-six percent of the US sample had not completed high school, and 50% were working full-time prior to incarceration.

### Procedures

The CAAPE (Comprehensive Addictions And Psychological Evaluation) as adapted for use in the UK is a fully structured interview that covers six Axis I and six Axis II conditions in addition to substance use disorders. It provides detailed indications for abuse or dependence for nine substance categories (alcohol, marijuana, cocaine, heroin, stimulants, sedatives, hallucinogens, inhalants, and a category for other substances or specific combinations).

The lead author (GYJ) interviewed all UK inmates at both institutions using the CAAPE shortly after each inmates' admission. This ensured that the diagnostic information was collected in a consistent and timely manner at each estate.

After the interviews were completed, staff entered the item responses into Excel spreadsheets. The Excel files were de-identified in that no names of identifiers were included that would identify an inmate to anyone outside of the facility. The anonymous data were then imported into SPSS (Statistics Package for the Social Science) for analyses.

Counsellors employed by the Minnesota Department of Corrections administered a structured interview using a computer prompted version of the SUDDS-IV (Substance Use Disorder Diagnostic Schedule-IV) at all adult correctional institutions in the state. The SUDDS-IV covers the same substance categories as the CAAPE and is also based on the DSM-IV diagnostic criteria. The computer program for the SUDDS-IV was loaded onto laptop computers used by the counsellors for their routine assessment of inmates and produced both a printed result for each inmate and a computer file of all cases evaluated. The data from each counsellor were uploaded to a composite file quarterly from the fall of 2000 through the end of 2002. De-identified data were used to produce quarterly and annual reports for the Department of Corrections and were also used for this study.

The 12-month timeframe for a positive abuse or dependence diagnosis in accordance with the DSM-IV-TR was the period prior to incarceration. Thus the diagnostic findings reported refer to the 12 months prior to incarceration for both populations.

Both the SUDDS-IV and CAAPE evaluations were part of routine evaluations, and the de-identified data with identifying information removed were provided with the approval of the respective institutions and agencies for statistical analyses of findings. Data used in the study are in compliance with the Helsinki Declaration regarding non-identified secondary data analysis and only produce aggregate information.

### Analyses

Diagnostic algorithms were developed according to DSM-IV/DSM-IV-TR criteria for both the CAAPE and SUDDS-IV to group items according to the seven dependence and four abuse criteria for each substance group. In order for an individual to be considered as having a possible diagnosis of alcohol or other drug dependence, findings for at least three of the seven dependence criteria had to be positive.

To further refine the diagnostic groupings, the subjects who met possible diagnoses for dependence were divided into three groups of low, medium, and high severity. Respectively, these groupings were defined as follows: those meeting only the minimal threshold of three positive dependence criteria, those meeting four or five of the criteria, and those positive on six or all seven of the criteria.

## Results

Alcohol dependence emerged as the most prevalent substance use disorder diagnosis for both the Grendon inmates (45%) and the Aylesbury inmates (86%). Overall, 75% of Grendon inmates and 97% of Aylesbury inmates met dependence criteria for some substance. For the US sample, alcohol also emerged as the most prevalent diagnosis (29%). The overall prevalence for any substance dependence is 52% for the US males.

The five most prevalent substance use disorder diagnoses for both UK facilities and the US state correctional system are listed in Table 1. With the exception of heroin, the young offenders in Aylesbury show higher dependence rates than the Grendon inmates. In both institutions, alcohol dependence is the most prevalent diagnosis for any specific substance. Also noteworthy is the fact that the general trend is for dependence on a given substance to be substantially more common than abuse only. The sole exception to this is for stimulants in the Aylesbury population where stimulant abuse is only slightly less common than stimulant dependence.

**Table 1:** Prevalence Rates for Substance Abuse and Dependence

**Substance**	**Grendon N = 118**		**Aylesbury N = 37**		**Minnesota N = 6,881**	
	Abuse	Dependence	Abuse	Dependence	Abuse	Dependence

Alcohol	3%	45%	2%	86%	16%	29%
Marijuana	3%	23%	5%	81%	13%	18%
Cocaine	3%	41%	8%	79%	4%	9%
Heroin	3%	27%	5%	24%	<1%	2%
Stimulants	4%	11%	22%	27%	4%	12%
Maximum Diagnosis *	3%	75%	0%	97%	21%	52%

* Maximum diagnosis refers to the proportion of cases with any dependence or abuse only diagnosis where a diagnosis of dependence for any substance overrides a diagnosis of abuse for another substance.

The prevalence rates for abuse and dependence are lower for the general US inmates, but the relative prevalence rates are consistent with those of the UK institutions in that alcohol dependence is the most prevalent dependence diagnosis and the majority of those with any dependence are dependent upon alcohol.

When severity is considered, Table 2 reveals that for the UK inmates the general trend is for those who meet at least minimal criteria for dependence to fall into the more severe range defined by those with positive findings for at least six of the seven DSM-IV/DSM-IV-TR dependence criteria. Of those with positive dependence diagnoses for alcohol, cocaine and heroin, the median number of positive dependence categories was six. For those with marijuana or stimulant dependence, the respective medians were five and four. With the exception of stimulants, half of those positive for dependence on a given substance were found to be positive on at least five of seven dependence criteria.

**Table 2:** Severity Levels for Dependence Among United Kingdome Inmates N = 155

Substance	No Diagnosis	Abuse	Low Severity Dependence	Moderate Dependence	Severe Dependence
Alcohol	41%	2%	6%	12%	39%
Marijuana	60%	3%	7%	17%	13%
Cocaine	48%	4%	5%	14%	29%
Heroin	70%	4%	2%	10%	14%
Stimulants	77%	8%	5%	7%	3%

Interestingly, of those manifesting alcohol, cocaine, or heroin dependence, over 60% were also positive for all four of the respective abuse criteria as well. None of the individuals qualifying for an abuse only diagnosis for any of the substances were positive on all four of their respective abuse criteria.

Table 3 provides the same severity analysis for the US inmates. Not surprisingly, the prevalence rates are considerably lower than that found in the UK facilities specializing in addressing inmates with behavioural health problems. However, two key findings hold for the Minnesota state prison inmate population. First, alcohol emerges as the most prevalent substance involved in dependence. Second, of those meeting dependence diagnosis for a given substance, the tendency is to be in the more severe ranges. Except for marijuana, the majority of those meeting dependence criteria for a given substance in the US sample are positive on at least six of the seven dependence criteria for that substance.

**Table 3:** Severity Levels for Dependence Among United States Inmates N = 6,881

Substance	No Diagnosis	Abuse	Low Severity Dependence	Moderate Dependence	Severe Dependence
Alcohol	54%	16%	5%	9%	16%
Marijuana	69%	13%	4%	7%	7%
Cocaine	87%	4%	1%	2%	6%
Heroin	98%	<1%	<1%	<1%	1%
Stimulants	85%	4%	1%	3%	7%

In both the UK and US, the overlap between alcohol and other drug dependence is considerable. Of the UK inmates dependent on a drug other than alcohol, 66% are also dependent on alcohol. For the US sample, 42% of those dependent on a drug are also dependent on alcohol, and an additional 10% are positive for alcohol abuse. Approximately 10% of inmates in both the UK and US samples are alcohol dependent in the absence of any other drug dependence.

In summary, the findings from the UK and US inmates are consistent in that alcohol abuse and dependence emerge as the most prevalent substance use disorders among inmates. Even among those with a drug dependence diagnosis, the majority also are positive for either alcohol dependence or abuse. Furthermore, those individuals with an alcohol dependence diagnosis typically are positive for at least six of the seven DSM-IV-TR criteria whether in the US or UK.

## Discussion

This study has a number of implications. Although correctional systems tend to focus more on drugs than alcohol, it is alcohol that is the most prevalent substance involved in dependence among prison inmates. Even when other drugs are involved, the likely probability is that the individual is also dependent upon alcohol. Therefore, any treatments designed for this population must take into account alcohol as well as other drugs in the design of the treatment programmes.

Many diversion and early release programs including drug courts in the US are restricted to non-violent offenders with drug use disorders. This restriction will exclude alcohol dependent individuals who have any assault or other technically violent offence even though alcohol dependent individuals may have a better prognosis than those dependent upon other drugs [11,12].

The analysis of severity for dependence suggests that dependence is a unique, prevalent, and distinct diagnosis as compared to abuse, which is compatible with previous findings [13]. Of those crossing the threshold for dependence as defined by the DSM-IV, the vast majority clearly exceed minimal criteria and most manifest syndromes involving at a minimum, five of the seven dependence criteria. Additionally, dependent individuals are typically positive on the majority of the four abuse criteria as well. Current results suggesting the unique nature of dependence are consistent with the work of Hasin and colleagues emphasizing the distinction between alcohol abuse and dependence [14] and the findings of Schuckit and colleagues regarding the differential prognoses of alcohol abuse vs. dependent individuals [15,16]. The greater prevalence of dependence relative to abuse in correctional populations is compatible with other general population surveys [17].

Limitations of the study are that the data are limited to just two institutions in the UK and one state in the US. Findings in other institutions in the UK may differ and it is possible that some differences might be found over time if data collection was continued. However, the findings that alcohol is the most prevalent substance involved in dependence diagnoses and that dependence for a given substance tends to be more prevalent than abuse is consistent with data from the US.

Limitations not withstanding, alcohol dependence is clearly a critical issue among inmates. Treatment efforts must address both alcohol and other drugs if we are to expect positive results.

## Competing interests

Gail Jones has no potential competing financial or other interests.

Norman Hoffmann is the author of the CAAPE and co-author of the SUDDS-IV from which the data in this article are derived. He receives a royalty from The Change Companies, the distributor of the instruments.

## Authors' contributions

GYJ conducted all clinical interviews, entered the anonymous information into an electronic spreadsheet, and assisted in the production of the manuscript. NGH conducted the analyses and assisted in the production of the manuscript. Both authors have read and approved the final manuscript.
